# Rebuttal to Correspondence on “Are Biobased Microfibers Less Harmful than Conventional Plastic Microfibers: Evidence from Earthworms”

**DOI:** 10.1021/acs.est.5c01575

**Published:** 2025-04-25

**Authors:** W. Courtene-Jones, F. De Falco, F. Burgevin, R. D. Handy, R. C. Thompson

**Affiliations:** † School of Biological and Marine Sciences, University of Plymouth, Drake Circus, Plymouth, Devon PL4 8AA, U.K.; ‡ School of Ocean Science, Bangor University, Anglesey LL59 5AB, U.K.; § School of Geography, Earth and Environmental Sciences, University of Plymouth, Drake Circus, Plymouth, Devon PL4 8AA, U.K.; ∥ Institute for Sustainability, Department of Chemistry, University of Bath, Bath BA2 7AY, U.K.

A recent commentary (Kogler et al. 2025)^1^ on our paper entitled “Are Biobased Microfibers Less Harmful than Conventional Plastic Microfibers: Evidence from Earthworms”^2^ makes assertions about our study which we clarify here. Their correspondence article also shows misunderstanding of the use of standardized ecotoxicity testing in relation to biobased/biodegradable plastics.

Kolger et al. (2025)^1^ raised a concern that because the Organisation for Economic Cooperation and Development (OECD) technical guidance (TG) 207^2^ is an acute screening test, it is not therefore appropriate to draw “*quantitative conclusions*” without more detailed testing in artificial soil. Our approach had multiple steps in the testing strategy: an acute exposure to derive dose–response curves for mortality following OCED TG 207,^3^ followed by chronic exposure in natural soil following the OECD TG 222 earthworm reproduction test^4^ with additional biochemical, histopathological, and behavioral end points to examine individual and likely population-level effects with respect to reproductive success and animal health. The OECD TG 207, like other OECD ecotoxicity tests, was written, validated (e.g., via interlaboratory testing), and approved by the international scientific community. So, under the Mutual Acceptance of Data principle,^5^ one should accept the results of the OECD TG 207, when it is conducted correctly, as we have done. In the context of the OECD scheme, the filter paper contact method in OECD TG 207 earthworm acute toxicity test^3^ constitutes a robust, initial assessment to identify chemicals of concern, preceding further testing. Of course, for research, the context often extends beyond individual standardized tests that are used commercially to provide hazard data to support environmental safety submissions to regulatory agencies. In addition to the growth, mortality, and reproduction assays specified in OECD TG 222^4^ for soil exposure, we included biochemical, histopathological, and behavioral end points, as these measurements provide mechanistic insights into the effect of pollutants on organisms, e.g., on cellular processes. It is also important to note that a negative (no addition of fibers) and a positive organic chemical control (used to confirm the responsiveness of the test systems) were included throughout our experiments. The inclusion of the positive control and the additional end points exceed the requirements in the OECD guidance, underscoring the robust approaches implemented and the weight of evidence reported. Similarly, OECD TG 207 and/or TG 222 have been widely used in published research, with additional end points to understand biological mechanisms, including particulate materials and fibers (e.g., refs (6−9)). Furthermore, our considerations on protocol amendments to earthworm tests for nanomaterials^10^ have recently been extended to plastics.^11^ Regarding the suggestion by Kogler et al. (2025)^1^ that an artificial soil should be used, we much prefer the environmental realism of a natural soil, notably the Lufa 2.2 soil that is widely used in earthworm studies (e.g., refs (12−14)).

Kogler et al. (2025)^1^ also state that it is not plausible for a substance to be toxic at low concentration, but not at the higher concentrations in an acute test. Such thinking is derived from solute chemistry and does not consider the bioaccessibility, bioavailability, or the behavior of the material in the exposure media. Microplastic particles, and other forms of particulate pollutants (e.g., nanoparticles, soot particles, sparingly soluble metal oxides), are colloids in water and do not behave like solutes (see Handy et al., 2008^15^ on colloid theory and ecotoxicity). For example, at lower concentrations, particulates may be more dispersed and therefore have a greater total surface area for bioavailability than at higher concentrations. At high particle number concentrations, aggregation behavior could greatly reduce bioaccessibility and therefore bioavailability and toxicity. For these reasons, the dose–response curve is not necessarily going to be like that for a solute, and the metrics used for dosimetry in particle toxicology have been extensively discussed (e.g., Hull et al. 2012^16^). It is, therefore, by no means implied that higher exposure automatically translates to greater harmful effects. In recognition of those uncertainties in the dose metric, the toxicity data presented in our study were reported as both particle number concentration and mass concentration.

In their comment, Kogler et al. (2025)^1^ suggest that for biodegradable materials, toxicity testing should be performed on the degradation products rather than the parent material. For historical reasons, polymers, including those made of plastics, are regulated in a different way than other chemicals. In the EU, polymers have been mostly classified as so-called “*polymers of low concern*” on the assumption of low toxicity and therefore not usually subject to the Registration, Evaluation, Authorisation, and Restriction of Chemicals (REACH) regulations, EC 1907/2006, unless they contain 2% or more of monomers that might behave as a solute and the polymer production exceed one tonne per annum. This view is being challenged by the scientific community (e.g., Groh et al. 2023^17^). The REACH regulations have recently been amended to include polymers from (micro)plastics (regulation EU 2023/2055), and this reporting will likely become mandatory during 2025. Regardless of historic regulatory requirements, it remains of key environmental relevance to determine the hazard of a product in the form in which it is released into the environment. In the case of fibers, and polymers used in this study, the application of sewage-derived biosolids directly introduces substantial quantities into terrestrial systems each year.^18,19^ Further, colored fibers, including those of biobased origins, are widely documented in the environment including in regions far from input sources, such as in deep-sea sediments^20^ and Arctic ice cores,^21^ indicating their persistence and the precedence for testing the particles themselves. Moreover, one must perform a study before the degradation products can be determined. Any degradation products emitted from the materials during our 56-days of chronic exposure (28 days adult exposure, with a further 28 days for fecundity assessments) would have been contained within the soil experimental system, with earthworms subsequently exposed to these. Due to the technical barriers of conducting such analytical chemistry of potentially multiple breakdown products and/or metabolites in complex environmental matrices, there are no requirements to measure these in OECD ecotoxicity test guidelines in the exposed organisms, and this was not practical in our study for the same reasons; nonetheless, the biological end points in a controlled laboratory experiment can confirm if exposure to a hazardous substance(s) has occurred. The evidence of tissue repair in the histology presented in our original article, the absence of glutathione depletion, and normal concentrations for most of the electrolytes measured, indicate that the animals were meeting the bioenergetic cost of toxicity.^2^ As stated in our original article, the aim was not to determine the detailed mechanisms of toxicity but to perform ecologically relevant experiments examining the individual and likely population level effects of the fibers. Inevitably, full mechanistic understanding will come after more experiments, with additional end points and approaches, beyond the scope of a single paper. It is also important to recognize that effects vary between species, and more complete understanding on toxicity of biobased materials such as these will require further testing with other species and in other environmental settings.

Finally, with respect to the appropriateness of the data analysis, our original article^2^ applied internationally validated, routine methods (i.e., OECD protocols), with levels of replication (*n* = 10 for filter paper contact experiment, *n* = 4 replicate vessels for chronic exposure) which are aligned with, or exceed, those recommended by the OECD and the scientific community.^22,23^ The level of replication and data assumptions (e.g., checked for abnormality, skewness) were appropriate for the statistical tests performed^24^ and are widely used in peer-reviewed studies,^13,25^ with results reported as statistically significant or not significant as in the paper.
